# Empagliflozin reduced long-term HbA1c variability and cardiovascular death: insights from the EMPA-REG OUTCOME trial

**DOI:** 10.1186/s12933-020-01147-9

**Published:** 2020-10-13

**Authors:** Antonio Ceriello, Anne Pernille Ofstad, Isabella Zwiener, Stefan Kaspers, Jyothis George, Antonio Nicolucci

**Affiliations:** 1 IRCCS MultiMedica, Milan, Italy; 2 Boehringer Ingelheim Norway KS, Asker, Norway; 3 Boehringer Ingelheim Pharma GmbH & Co. KG, Ingelheim, Germany; 4 Boehringer Ingelheim International GmbH, Ingelheim, Germany; 5 Center for Outcomes Research and Clinical Epidemiology, Pescara, Italy

**Keywords:** Type 2 diabetes, Glucose variability, Empagliflozin, Cardiovascular death

## Abstract

**Background:**

Glucose variability has been associated with cardiovascular outcomes in type 2 diabetes, however, the interplay between glucose variability, empagliflozin and cardiovascular death has not been explored. In the EMPA-REG OUTCOME trial, empagliflozin reduced the risk of cardiovascular death by 38%. We explore post-hoc the association between HbA1c variability and cardiovascular death, and the potential mediating effects of HbA1c variability on empagliflozin’s cardiovascular death reductions.

**Methods:**

In total, 7,020 patients with type 2 diabetes and established cardiovascular disease received placebo, empagliflozin 10 mg or 25 mg. We defined within-patient HbA1c variability as standard deviation, coefficient of variation and range of HbA1c measurements (%) post-baseline. First, we compared HbA1c variability until week 28 and 52 by Wilcoxon tests. We explored the association between cardiovascular death and HbA1c variability in placebo and pooled empagliflozin arms separately with landmark analyses at week 28 and 52, and additionally with HbA1c variability as a time-dependent co-variate. We used Cox regression models adjusted for baseline risk factors including changes in HbA1c from baseline to week 12, and the interaction term HbA1c variability* treatment.

**Results:**

HbA1c variability was lower with empagliflozin compared to placebo. In all Cox analyses, high HbA1c variability increased the risk for cardiovascular death in both treatment arms with no interaction with treatment: e.g. an increase in HbA1c variability of one unit for the standard deviation at week 28 was associated with a subsequent increased risk of CV death with HRs of 1.97 (95% CI 1.36, 2.84) and 1.53 (1.01, 2.31) in the placebo and empagliflozin groups, separately, interaction p-value 0.3615.

**Conclusions:**

HbA1c variability was reduced by empagliflozin and high values of HbA1c variability were associated with an increased risk of cardiovascular death. Empagliflozin’s reduction in cardiovascular death did not appear to be mediated by reductions in HbA1c variability.

ClinicalTrials.gov number, NCT01131676

## Background

People with type 2 diabetes are at increased risk of macrovascular complications and events including cardiovascular death. Although high levels of glycemia are associated with increased risk of these complications, the effect from intensive glucose lowering is at best modest [1]. Recently, long-term glucose variability in terms of variability of HbA1c and fasting blood glucose has been shown to be associated with cardiovascular complications and mortality in diabetes. These associations are suggested by several epidemiological studies [2] as well as by a cohort study demonstrating that patients with type 2 diabetes with a higher glucose variability as measured by the coefficient of variation of glucose had an unfavourable metabolic profile and increased risk of developing micro- and macrovascular complications and mortality [3]. These results are in line with post-hoc analyses of four major trials in type 2 diabetes; The Action in Diabetes and Vascular Disease: Preterax and Diamicron Modified Release Controlled Evaluation (ADVANCE) trial [4], the Veterans Affairs Diabetes Trial (VADT) [5], The Trial Comparing Cardiovascular Safety of Insulin Degludec vs Insulin Glargine in Patients with Type 2 Diabetes at High Risk of Cardiovascular Events (DEVOTE), and the Action to Control Cardiovascular Disease in Diabetes (ACCORD) trial [6, 7]. These trials demonstrated that day-to day glucose variability is associated with mortality (DEVOTE), that long-term glucose variability is associated with an expanded outcome of major cardiovascular adverse events (VADT), and that visit-to-visit-variability is associated with cardiovascular events and mortality (ADVANCE, ACCORD).

The EMPA-REG OUTCOME trial explored the cardiovascular safety and effects of the sodium-glucose-co-transporter 2 (SGLT2) inhibitor empagliflozin in patients with type 2 diabetes and established cardiovascular disease [8]. Empagliflozin significantly reduced the primary composite outcome of cardiovascular death, nonfatal myocardial infarction and nonfatal stroke, primarily driven by a reduction in the risk of cardiovascular death of 38% as compared to standard of care [9]. This reduction in cardiovascular death was shown to be independent from glycemic control at baseline and during the trial [10], and was not attenuated by incident hypoglycaemia [11]. Moreover, hypoglycaemia was associated with an increased risk of hospitalisation for heart failure, but the risk of hypoglycaemia was not increased with empagliflozin [11]. The role of HbA1c variability in the reductions of cardiovascular death with empagliflozin has not yet been explored.

The aim of these post-hoc analyses was to evaluate the impact of empagliflozin on HbA1c variability in the EMPA-REG OUTCOME trial, and furthermore explore whether HbA1c variability is associated with cardiovascular death or mediates the treatment effect from empagliflozin on cardiovascular death in a population with type 2 diabetes and cardiovascular disease.

## Methods

### Study design

The design of EMPA-REG OUTCOME has been described previously [8]. Briefly, the study population comprised patients with type 2 diabetes, established cardiovascular disease, HbA1c 7.0–9.0% for drug-naïve patients and 7.0–10.0% for those on stable glucose-lowering therapy, and estimated glomerular filtration rate (eGFR) (MDRD [Modification of Diet in Renal Disease] equation) ≥ 30 mL/min/1.73 m^2^. Patients were randomized 1:1:1 to receive empagliflozin 10 mg, empagliflozin 25 mg, or placebo in addition to standard of care. Throughout the trial (or after week 12 for glucose lowering medication), investigators were encouraged to treat cardiovascular risk factors to achieve optimal standard of care according to local guidelines. The trial was to continue until ≥ 691 patients had experienced an adjudicated event included in the primary outcome.

### Outcomes

Definitions of the major clinical outcomes in the EMPA-REG OUTCOME trial have been published [9]. All cardiovascular outcome events and deaths were prospectively adjudicated by two Clinical Events Committees (for cardiac and neurological events). In the current analyses we explore cardiovascular death.

### HbA1c variability definitions

In these post-hoc analyses, we defined HbA1c variability as the intra-individual standard deviation (SD), coefficient of variation (CV) as well as range of all measurements of HbA1c (given in %) [2]. For the comparison of glucose variability between treatment arms and for the Landmark analyses at week 28 and 52, HbA1c variability was calculated using all post-baseline measurements up until and including week 28 (maximum two HbA1c measurements) and 52 (maximum four HbA1c measurements) (Additional file 1: Table S1). To capture HbA1c variability during the full trial (also after week 28 and 52), HbA1c variability was modelled as a time-dependent co-variate with linear interpolation between visits (starting with pre-treatment HbA1c variability). Additional file 1: Table S1 shows the schedule for HbA1c measurements during the trial.

We also calculated the variability of fasting blood glucose (measured in mg/dL) in the same manner as for HbA1c, up until week 28 and week 52, and performed Landmark analyses at these time points.

### Statistical analyses

For all analyses, patients with too few HbA1c measurements to calculate variability were excluded. We used the Wilcoxon test to compare HbA1c variability in the pooled empagliflozin arms vs. placebo at week 28 and 52. To explore if HbA1c variability is a prognostic factor for cardiovascular death, we performed Landmark analyses exploring the association of HbA1c variability until week 28 or 52, and the risk of subsequent cardiovascular death. As a sensitivity analysis, we included the time-dependent variable of HbA1c variability as a continuous variable in the Cox models to explore how HbA1c variability during the trial was associated with cardiovascular death. As a second sensitivity analysis we performed similar analyses for fasting blood glucose variability as for HbA1c variability.

To further explore the relationship between HbA1c variability and cardiovascular death, we examined HRs for cardiovascular death across quintiles of HbA1c variability as a time-dependent categorical covariate. The risk of each of the four upper quintiles was compared with the first quintile (i.e. assuming no trend). The cut-offs for the quintiles were derived from the distribution of per-subject HbA1c variability at each planned visit in the overall patient population (thus, generally including more than 1 value for HbA1c variability per subject and pooling both treatment groups). The quintile category for each individuals’ specific variability measure was time-dependent and was assigned based on the HbA1c values from earlier time points. Finally, to explore whether the reduced risk of cardiovascular death is potentially (at least in part) mediated by HbA1c variability reductions in empagliflozin treated patients, we performed Cox-regression analysis with and without adjustment for quintiles of HbA1c variability as a time-dependent co-variate. All analyses of associations between HbA1c variability and outcomes were undertaken in the pooled empagliflozin vs. placebo groups, and based on Cox proportional hazard models that included terms for baseline age, sex, baseline HbA1c (categorized), baseline body mass index (categorized), baseline eGFR (categorized), geographical region, treatment, HbA1c variability and HbA1c variability by treatment interaction, as well as change in HbA1c from baseline to week 12. For the analyses assessing the association of fasting blood glucose variability to cardiovascular death, fasting blood glucose variability and fasting blood glucose variability by treatment interaction terms were included instead of the respective terms with HbA1c variability, and the change from baseline to week 4 in fasting blood glucose replaced the change in HbA1c. We did not perform any corrections for multiple comparisons. Analyses were performed with SAS version 9.4

## Results

Characteristics of the study population have been published previously [9]. Briefly, in the overall study population (7020 patients), there were 28.5% females, and mean age at baseline was 63.1 years, mean HbA1c was 8.07% (64.7 mmol/mol), fasting blood glucose 152.9 mg/dL, and 57.1% had a diabetes duration of more than 10 years. Median follow-up time was 3.1 years. In the Landmark analyses at week 28, 450 patients were excluded due to lack of sufficient number of HbA1c measurements to calculate HbA1c variability. For the Landmark analyses at week 52 and for the analyses with HbA1c variability as a time-dependent co-variate, the number of patients excluded were 604 and 342, respectively.

### Empagliflozin’s impact on glucose variability

HbA1c variability by all three definitions (SD, CV or range) were significantly lower at week 28 and 52 with empagliflozin vs. placebo (Table 1).

**Table 1:** HbA1c (%) variability in the pooled empagliflozin and placebo arms separately, at week 28 and 52

	Number of patients		Standard deviation Median (IQR)		Coefficient of variation Median (IQR)		Range Median (IQR)	
	Week 28	Week 52	Week 28	Week 52	Week 28	Week 52	Week 28	Week 52
Placebo	2171	2112	0.3 (0.1, 0.6)	0.4 (0.3, 0.7)	3.9 (1.9, 6.8)	5.4 (3.6, 8.2)	0.4 (0.2, 0.8)	1.0 (0.6, 1.4)
Empagliflozin	4400	4304	0.2 (0.1, 0.4)	0.3 (0.2, 0.5)	3.1 (1.2, 5.8)	4.4 (3.0, 6.6)	0.3 (0.1, 0.6)	0.7 (0.5, 1.1)
p-value*			< 0.0001	< 0.0001	< 0.0001	< 0.0001	< 0.0001	< 0.0001

p-value for difference between placebo and pooled empagliflozin arms. Only patients with at least two post-baseline HbA1c measurements up until week 28 or 52, respectively, are included

* From Wilcoxon test

*IQR* Inter-quartile-range

### HbA1c variability as a prognostic factor for cardiovascular death

**Fig. 1:**
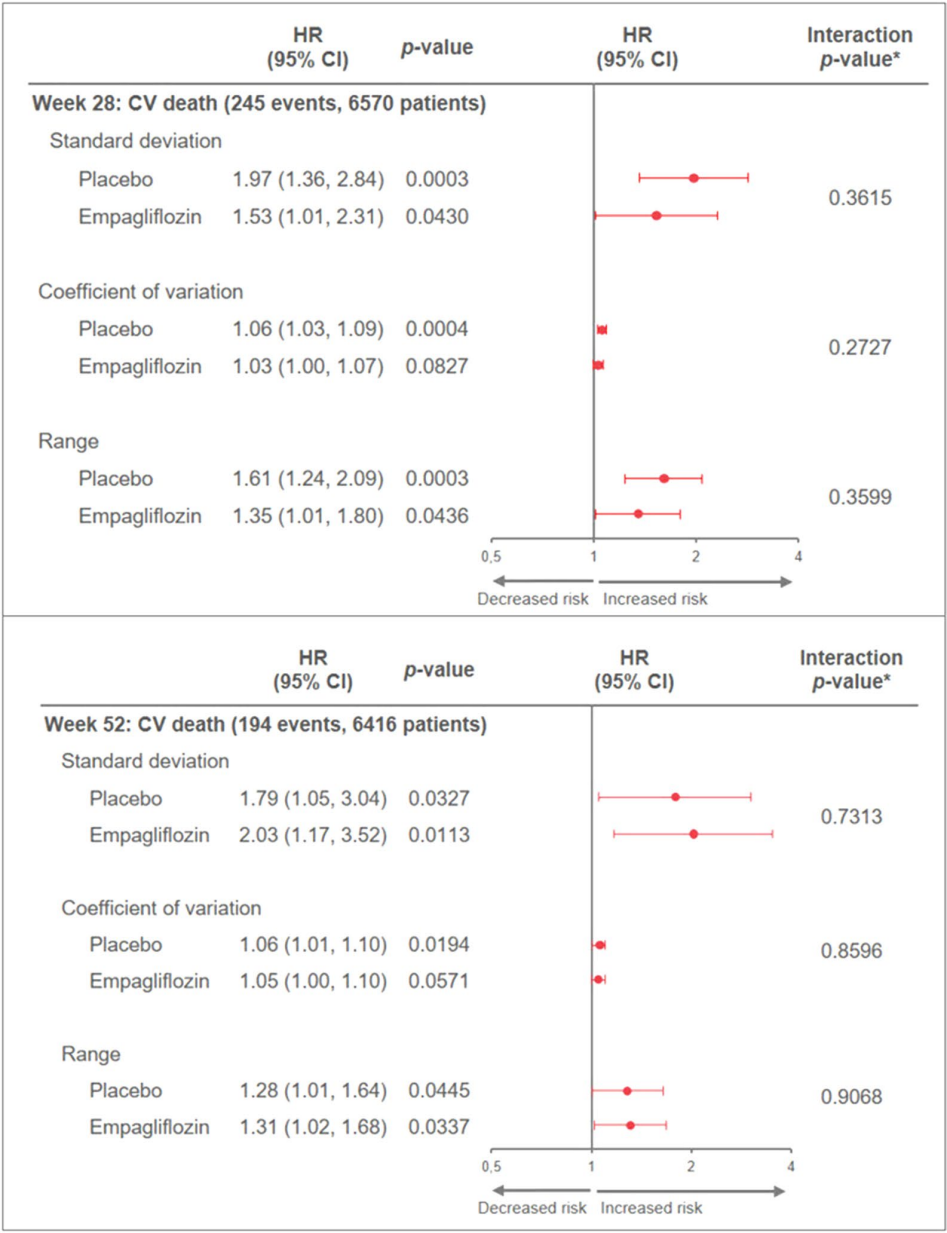
Association of HbA1c variability at week 28 (**a**) and 52 (**b**) and subsequent CV deaths (Landmark analysis) in the treatment groups separately. Only patients still at risk for CV death at week 28 (week 52, resp.) and with at least 2 post-baseline HbA1C measurements up to week 28 (week 52, resp.) included. HRs are for a 1-unit (%) increase in HbA1c variability. *CV* cardiovascular, *HbA1c* glycated hemoglobin, *LM* landmark. Cox models include: age, sex, Hba1c, BMI, eGFR, geographic region, treatment, change in HbA1c from baseline to week 12, HbA1c variability up to week 28 (week 52, resp.) and its interaction with treatment. *HbA1c variability*treatment interaction

For all analyses, results were similar irrespective of which measure of variability (SD, CV or range) was used. One unit (%) increase in HbA1c variability at week 28 and 52 was associated with subsequent increased risk of cardiovascular death in the Landmark analyses, with similar associations in both treatment arms [p for HbA1c variability*treatment interaction p > 0.05 (Fig. 1a and b)]

The sensitivity analyses exploring the association between HbA1c variability during the trial and cardiovascular death with HbA1c variability as a time-dependent co-variate were in line with the 28 and 52 week Landmark analyses (Additional file 1: Figure S1).

The analyses exploring the association of quintiles of HbA1c variability and cardiovascular death showed no clear linear association but generally confirmed a (non-strictly) monotone trend with the highest risk for cardiovascular death seen in the highest quintile of HbA1c variability. (Additional file 1: Figure S2).

The analyses exploring the association between fasting blood glucose variability and cardiovascular death were in line with the analyses using HbA1c variability (Additional file 1: Figures S4 A and B, and S5).

### HbA1c variability and treatment effect of empagliflozin on cardiovascular death

In all analyses there were no significant interactions between HbA1c variability and treatment with all interaction p-values being > 0.05 (Fig. 1a and b, Additional file 1: Figures S1 and S3), suggesting similar effects of HbA1c variability irrespective of treatment group.

There were only small differences in the estimated treatment effect of empagliflozin vs placebo on cardiovascular death when comparing analyses with and without adjustment for quintiles of HbA1c variability. This indicates that reductions in HbA1c variability do not notably mediate empagliflozin’s treatment effect on cardiovascular death (Additional file 1: Figure S3).

## Discussion

This study demonstrates that empagliflozin reduced long-term HbA1c variability in the EMPA-REG OUTCOME trial, and furthermore that high HbA1c variability early (i.e. within first year of treatment) and during the trial is associated with increased risk of cardiovascular death. Empagliflozin reduces the risk of cardiovascular death, but this effect does not seem to be mediated by the reduction in HbA1c variability.

### The association between HbA1c variability and cardiovascular outcomes

Mounting evidence demonstrates that glucose variability is associated with cardiovascular complications, particularly cardiovascular death [2-6], not only in diabetes but also in people without diabetes [12-14], and our study adds to this body of evidence. Interestingly, a small study in patients with type 2 diabetes undergoing percutaneous coronary intervention found that while HbA1c identified patients at higher thrombotic risk, the highest diagnostic accuracy for high platelet reactivity seemed to be achieved by combining glucose variability and HbA1c [15]. Our analyses, adjusting for baseline HbA1c levels, support that using a combination of HbA1c and HbA1c variability may improve the risk assessment of patients with type 2 diabetes.

Moreover, our sensitivity analyses assessing HbA1c variability as a time-dependent co-variate and assessing the association of fasting blood glucose variability to cardiovascular death showed results in line with our main analyses, supporting our conclusions.

### SGLT2 inhibitors’ impact on glucose variability

At the same time, SGLT-2 inhibitors, including empagliflozin, reduce glucose variability in both type 2 and type 1 diabetes [16-18]. In our study, we found that empagliflozin significantly reduced HbA1c variability as compared to placebo, but this reduction did not seem to be a mediator of the reduction in cardiovascular death with empagliflozin. Published analyses demonstrate that the reduction in cardiovascular death with empagliflozin is independent of glucose control at baseline and during the trial [10], indicating that the reduction was not driven by control of glucose. In line with this, a mediation analysis found changes in markers of plasma volume (haematocrit) to be the strongest mediator of cardiovascular death reductions whereas measures of glycaemia seemed to have only modest mediating effects [19]. Moreover, although the blood glucose lowering effect of empagliflozin diminishes with declining eGFR [20], the cardioprotective effects are consistent down to eGFR 30 ml/min/ 1.73 m^2^ [21].

On the other hand, intriguingly, it has been reported that in diabetic rats the reduction of glucose variability with dapagliflozin is associated with a reduction of early atherosclerosis of the vessel walls [22]. The impact of glucose variability on diabetic complications has been linked to an increase of oxidative stress generation [23], produced by an increase of free radicals accompanied by an inadequate antioxidant response of the cells [24, 25]. In diabetes, oxidative stress plays a key role in favouring the appearance of cardiovascular complications [26]. In patients with type 2 diabetes, dapagliflozin reduced glucose variability as well as oxidative stress [27], while it has been reported in an animal model of diabetes that empagliflozin can reduce oxidative stress generation [28]. Therefore, although not detected in our analyses, we believe that the role of reducing HbA1c variability in the beneficial effects of empagliflozin on cardiovascular death remains to be fully elucidated.

### Glucose variability’s role in clinical practice

Our findings of an association between HbA1c variability and cardiovascular death in the EMPA-REG OUTCOME trial also supports the possible role for glucose variability in the complications of diabetes [2]. The analyses exploring the association of quintiles of HbA1c variability and cardiovascular death suggested that the assumption of a continuous effect of increases in HbA1c variability (the higher the HbA1c variability, the higher the risk for cardiovascular death) underlying the previous analyses might not hold true, but rather indicate that there could be thresholds of HbA1c variability after which the risk of cardiovascular death increases (Additional file 1: Figure S2). This also suggests that the overall effect as seen in these analyses might in part be driven by patients with a rather high HbA1c variability (fifth and forth quintile).

This is important since glucose variability is emerging as a new challenge in the management of diabetes and is quickly moving from being just a scientific topic to clinical practice [29]. The increased availability of new type 2 diabetes treatments that impact glucose variability, and the increased availability of new tools to measure glucose variability, certainly favour the clinician to address this new aspect of diabetes management. Empagliflozin is on the list of drugs which potentially can reduce glucose variability, at the same time improving prognosis by reducing the risk of cardiovascular death as well as heart failure and renal outcomes (30, 31).

Our analyses have some limitations. These were post-hoc analyses from the EMPA-REG OUTCOME trial that was designed to explore the long-term cardiovascular safety and effects of empagliflozin. Thus, there were no daily measurements of glucose, and HbA1c was measured relatively infrequently (Additional file 1: Table S1). Moreover, HbA1c as a marker of glycaemic control has several limitations and new parameters such as time in range are emerging. Continuous blood glucose monitoring was not done in EMPA-REG OUTCOME and time in range therefore not captured. The study was powered to assess the effects of empagliflozin on the primary endpoint 3-point—major adverse cardiovascular events and was not powered to explore the effects on cardiovascular death alone. The strengths of our analyses are the long observation time with a high number of, and adjudication of, cardiovascular deaths. Furthermore, our analyses of three different definitions of HbA1c variability as well as fasting blood glucose variability confirms the robustness of our results.

## Conclusions

Our analyses demonstrate that empagliflozin reduces HbA1c variability as compared to standard of care in patients with type 2 diabetes and cardiovascular disease. High HbA1c variability is furthermore associated with higher risk of cardiovascular death, but reductions in HbA1c variability do not notably mediate empagliflozin’s treatment effect of reducing cardiovascular death. A possible explanation for this is that the effects of empagliflozin on cardiovascular death may be related to changes in sodium, water and energy metabolism rather than to effects on glucose. Our findings from the EMPA-REG OUTCOME trial, anyhow, add to the body of evidence around HbA1c variability which is an emerging aspect of type 2 diabetes treatment also in the clinical setting. Our results are important particularly considering the increased use of SGLT2 inhibitors in recent years.

## Supplementary information

**Additional file 1.** Additional figures and table.

## Data Availability

The sponsor of the EMPA-REG OUTCOME Trial (Boehringer Ingelheim) is committed to responsible sharing of clinical study reports, related clinical documents, and patient level clinical study data. Researchers are invited to submit inquiries via the following website: https://trials.boehringer-ingelheim.com

## Abbreviations

ACCORD: And the Action to Control Cardiovascular Disease in Diabetes trial
ADVANCE: The Action in Diabetes and Vascular Disease: Preterax and Diamicron Modified Release Controlled Evaluation
CV: Coefficient of variation
DEVOTE: The Trial Comparing Cardiovascular Safety of Insulin Degludec vs Insulin Glargine in Patients with Type 2 Diabetes at High Risk of Cardiovascular Events
eGFR: Estimated glomerular filtration rate
MDRD: Modification of Diet in Renal Disease
SD: Standard deviation
SGLT2: Sodium-glucose transporter 2
VADT: The Veterans Affairs Diabetes Trial

## References

[1] Turnbull FM, Abraira C, Anderson RJ, Byington RP, Chalmers JP, Duckworth WC (2009). Intensive glucose control and macrovascular outcomes in type 2 diabetes. Diabetologia.

[2] Ceriello A, Monnier L, Owens D (2019). Glycaemic variability in diabetes: clinical and therapeutic implications. The lancet Diabetes & endocrinology.

[3] Slieker RC, van der Heijden A, Nijpels G, Elders PJM, Hart LM, Beulens JWJ (2019). Visit-to-visit variability of glycemia and vascular complications: the Hoorn Diabetes Care System cohort. Cardiovasc Diabetol..

[4] Hirakawa Y, Arima H, Zoungas S, Ninomiya T, Cooper M, Hamet P (2014). Impact of visit-to-visit glycemic variability on the risks of macrovascular and microvascular events and all-cause mortality in type 2 diabetes: the ADVANCE trial. Diabetes Care.

[5] Zhou JJ, Schwenke DC, Bahn G, Reaven P (2018). Glycemic variation and cardiovascular risk in the veterans affairs diabetes trial. Diabetes Care.

[6] Zinman B, Marso SP, Poulter NR, Emerson SS, Pieber TR, Pratley RE (2018). Day-to-day fasting glycaemic variability in DEVOTE: associations with severe hypoglycaemia and cardiovascular outcomes (DEVOTE 2). Diabetologia.

[7] Ceriello A. Commentary on: Glucose Variability and Diabetic Complications: Is It Time to Treat? Diabetes Care. 2020 **(In press)**. 10.2337/dci20-0012 32434893

[8] Zinman B, Inzucchi SE, Lachin JM, Wanner C, Ferrari R, Fitchett D (2014). Rationale, design, and baseline characteristics of a randomized, placebo-controlled cardiovascular outcome trial of empagliflozin (EMPA-REG OUTCOME). Cardiovasc Diabetol.

[9] Zinman B, Wanner C, Lachin JM, Fitchett D, Bluhmki E, Hantel S (2015). Empagliflozin, cardiovascular outcomes, and mortality in type 2 diabetes. N Engl J Med..

[10] Inzucchi SE, Kosiborod M, Fitchett D, Wanner C, Hehnke U, Kaspers S (2018). Improvement in cardiovascular outcomes with empagliflozin is independent of glycemic control. Circulation.

[11] Fitchett D, Inzucchi SE, Wanner C, Mattheus M, George JT, Vedin O (2020). Relationship between hypoglycaemia, cardiovascular outcomes, and empagliflozin treatment in the EMPA-REG OUTCOME® trial. Eur Heart J.

[12] Echouffo-Tcheugui JB, Zhao S, Brock G, Matsouaka RA, Kline D, Joseph JJ (2019). Visit-to-visit glycemic variability and risks of cardiovascular events and all-cause mortality: the ALLHAT Study. Diabetes Care.

[13] Kim MK, Han K, Park YM, Kwon HS, Kang G, Yoon KH (2018). Associations of variability in blood pressure, glucose and cholesterol concentrations, and body mass index with mortality and cardiovascular outcomes in the general population. Circulation.

[14] Ghouse J, Skov MW, Kanters JK, Lind B, Isaksen JL, Blanche P (2019). Visit-to-visit variability of hemoglobin a1c in people without diabetes and risk of major adverse cardiovascular events and all-cause mortality. Diabetes Care.

[15] Nusca A, Tuccinardi D, Proscia C, Melfi R, Manfrini S, Nicolucci A (2019). Incremental role of glycaemic variability over HbA1c in identifying type 2 diabetic patients with high platelet reactivity undergoing percutaneous coronary intervention. Cardiovasc Diabetol.

[16] Henry RR, Strange P, Zhou R, Pettus J, Shi L, Zhuplatov SB (2018). Effects of dapagliflozin on 24-hour glycemic control in patients with type 2 diabetes: a randomized controlled trial. Diabetes Technol Therap.

[17] Rodbard HW, Peters AL, Slee A, Cao A, Traina SB, Alba M (2017). The effect of canagliflozin, a sodium glucose cotransporter 2 inhibitor, on glycemic end points assessed by continuous glucose monitoring and patient-reported outcomes among people with type 1 diabetes. Diabetes Care.

[18] Famulla S, Pieber TR, Eilbracht J, Neubacher D, Soleymanlou N, Woerle HJ (2017). Glucose exposure and variability with empagliflozin as adjunct to insulin in patients with type 1 diabetes: continuous glucose monitoring data from a 4-week, randomized, placebo-controlled trial (EASE-1). Diabetes Technol Therap.

[19] Inzucchi SE, Zinman B, Fitchett D, Wanner C, Ferrannini E, Schumacher M (2018). How does empagliflozin reduce cardiovascular mortality? Insights from a mediation analysis of the EMPA-REG OUTCOME Trial. Diabetes Care.

[20] Cherney DZI, Cooper ME, Tikkanen I, Pfarr E, Johansen OE, Woerle HJ (2018). Pooled analysis of Phase III trials indicate contrasting influences of renal function on blood pressure, body weight, and HbA1c reductions with empagliflozin. Kidney Int.

[21] Wanner C, Lachin JM, Inzucchi SE, Fitchett D, Mattheus M, George J (2018). Empagliflozin and Clinical Outcomes in Patients With Type 2 Diabetes Mellitus, Established Cardiovascular Disease, and Chronic Kidney Disease. Circulation.

[22] Stelmaszyk A, Wesolowska A, Pomieczynska K, Iskakova S, Frydrychowicz M, Dworacki G (2018). The impact of dapagliflozin on glucose excursions related to early proatherogenic derangement in the aortic wall. Saudi Pharm J.

[23] Ceriello A, Ihnat M (2010). Oxidative stress is, convincingly, the mediator of the dangerous effects of glucose variability. Diabet Med.

[24] La Sala L, Mrakic-Sposta S, Micheloni S, Prattichizzo F, Ceriello A (2018). Glucose-sensing microRNA-21 disrupts ROS homeostasis and impairs antioxidant responses in cellular glucose variability. Cardiovasc Diabetol.

[25] La Sala L, Cattaneo M, De Nigris V, Pujadas G, Testa R, Bonfigli AR (2016). Oscillating glucose induces microRNA-185 and impairs an efficient antioxidant response in human endothelial cells. Cardiovasc Diabetol.

[26] Ceriello A, Testa R, Genovese S (2016). Clinical implications of oxidative stress and potential role of natural antioxidants in diabetic vascular complications. Nutr Metab Cardiovasc Dis.

[27] Li FF, Gao G, Li Q, Zhu HH, Su XF, Wu JD (2016). Influence of dapagliflozin on glycemic variations in patients with newly diagnosed type 2 diabetes mellitus. J Diabetes Res.

[28] Oelze M, Kroller-Schon S, Welschof P, Jansen T, Hausding M, Mikhed Y (2014). The sodium-glucose co-transporter 2 inhibitor empagliflozin improves diabetes-induced vascular dysfunction in the streptozotocin diabetes rat model by interfering with oxidative stress and glucotoxicity. PLoS ONE.

[29] Raz I, Riddle MC, Rosenstock J, Buse JB, Inzucchi SE, Home PD (2013). Personalized management of hyperglycemia in type 2 diabetes: reflections from a Diabetes Care Editors' Expert Forum. Diabetes Care.

[30] Fitchett D, Zinman B, Wanner C, Lachin JM, Hantel S, Salsali A (2016). Heart failure outcomes with empagliflozin in patients with type 2 diabetes at high cardiovascular risk: results of the EMPA-REG OUTCOME(R) trial. Eur Heart J.

[31] Wanner C, Inzucchi SE, Lachin JM, Fitchett D, von Eynatten M, Mattheus M (2016). Empagliflozin and Progression of Kidney Disease in Type 2 Diabetes. N Engl J Med.

