# Supplementary material for: Empagliflozin reduced long-term HbA1c variability and cardiovascular death: insights from the EMPA-REG OUTCOME trial
Source: Cardiovasc Diabetol. 2020 Oct 13;19:176. doi: 10.1186/s12933-020-01147-9 (PMC7556977; doi:10.1186/s12933-020-01147-9)
Supplement: Supplementary file 1 — Additional file 1. Additional figures and table. [file 12933_2020_1147_MOESM1_ESM.docx]

Additional materials

“Empagliflozin reduces long-term HbA1c variability and cardiovascular death: insights from the EMPA-REG OUTCOME trial”

**Additional file 1: Table S1:** overview of the timing of the HbA1c and fasting blood glucose -measurements during the trial

| Trial Period | Screening | Placebo-run in |  |  |  |  |  |  |  |  |
| --- | --- | --- | --- | --- | --- | --- | --- | --- | --- | --- |
| Study week | -3 | -2 | * | 4 | 12 | 28 | 40 | 52 | Every 14 w after visit 10 to final visit | End of study visit |
| HbA1c | X |  | X |  | X | X | X | X | X | X |
| Fasting blood glucose |  | X | X | X | X | X |  | X | X | X |

*Day of randomization

**Additional file 1:Figure S1:** Association of HbA1c variability by HbA1c as a continuous time-dependent co-variate and CV death in the treatment groups separately, by landmark analysis starting at week 12


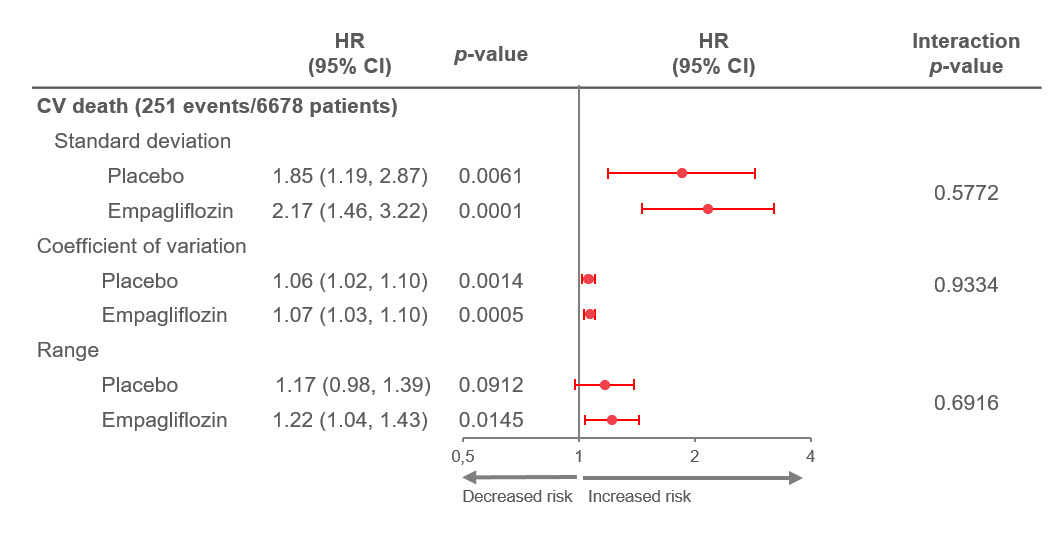


Only patients with least 2 pre-treatment and 2 post-baseline HbA1C measurements included. HRs are for a 1-unit (%) increase in HbA1c variability.

CV, cardiovascular; HbA1c, glycated hemoglobin.

Cox models include: baseline age, sex, baseline Hba1c, baseline BMI, baseline eGFR, geographic region, treatment, change in HbA1c from baseline to week 12, HbA1c variability as time-dependent covariate and its interaction with treatment.

* HbA1c variability*treatment interaction

**Additional file 1:Figure S2 A and B:** Association between quintiles of HbA1c variability as a time-dependent co-variate and CV death in the placebo (A) and pooled empagliflozin (B) groups (total n=6678), by landmark analysis starting at week 12. CI; confidence interval


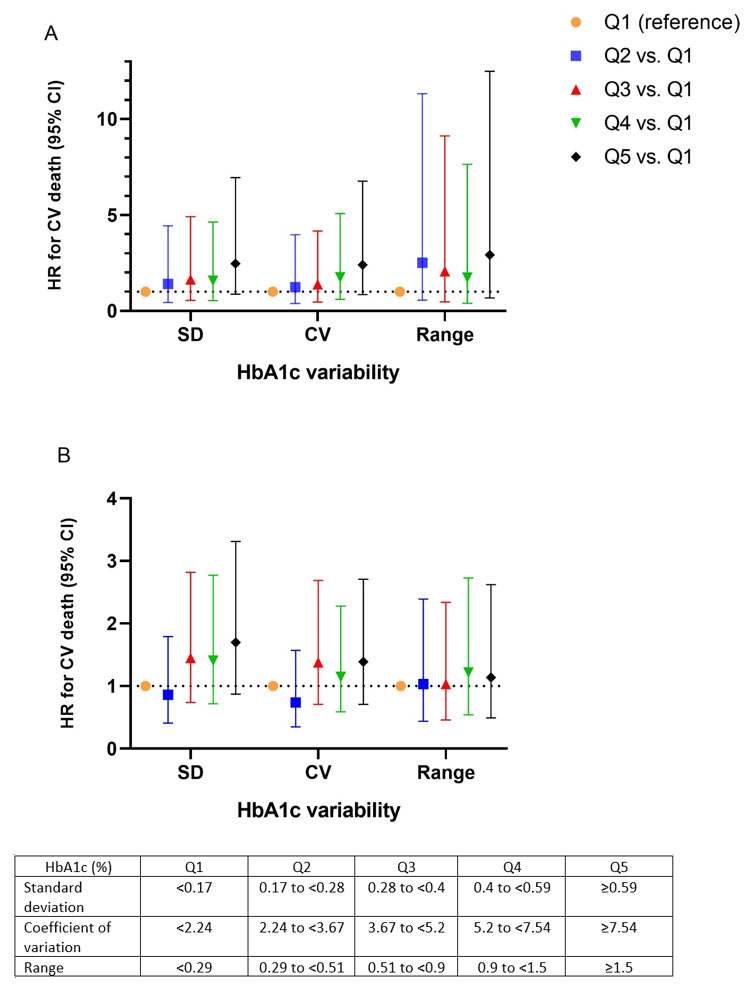


Only patients with least 2 pre-treatment and 2 post-baseline HbA1C measurements included. HRs are compared to Q1. CV, cardiovascular; HbA1c, glycated hemoglobin.

Cox model includes: baseline age, sex, baseline Hba1c, baseline BMI, baseline eGFR, geographic region, treatment, change in HbA1c from baseline to week 12, quintiles of HbA1c variability as time-dependent covariate and its interaction with treatment.

**Additional file 1:Figure S3:** Treatment effects of empagliflozin vs placebo on cardiovascular death, landmark analysis starting at week 12. Analyses adjusted for the primary model alone*, and adjusted for the primary model plus quintiles of HbA1c variability as a time-dependent covariate, to explore the mediation effects of HbA1c variability on the reduction in cardiovascular death.


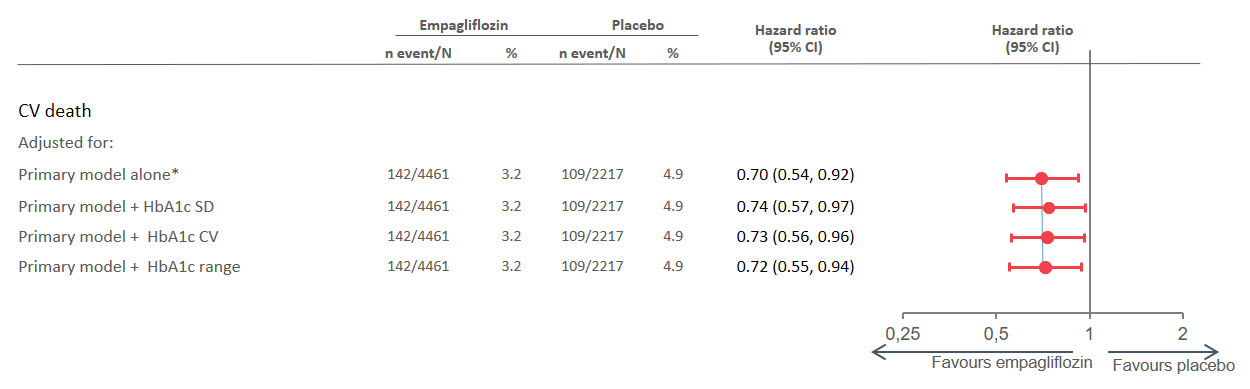


Only patients with least 2 pre-treatment and 2 post-baseline HbA1C measurements included.

* Primary Cox model includes terms for baseline age, sex, baseline eGFR, baseline BMI, baseline HbA1c, region, change in HbA1c from baseline to week 12, treatment.

**Additional file 1:Figure S4 A and B:** Association of fasting blood glucose variability at week 28 (a) and 52 (b) and subsequent CV deaths (Landmark analysis) in the treatment groups separately.


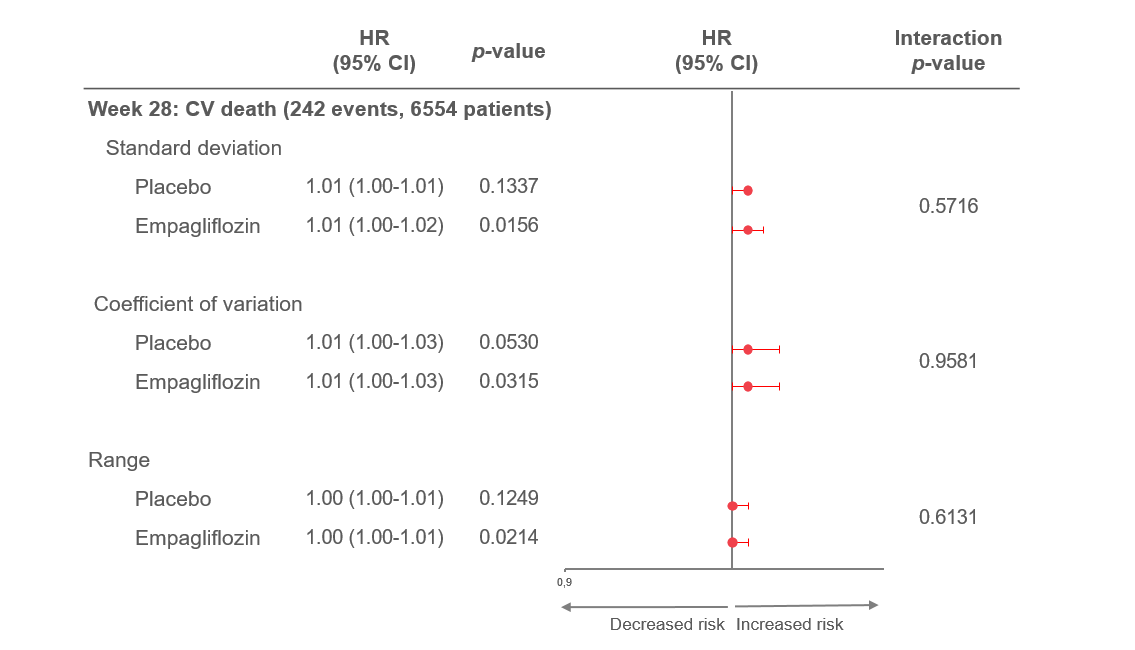


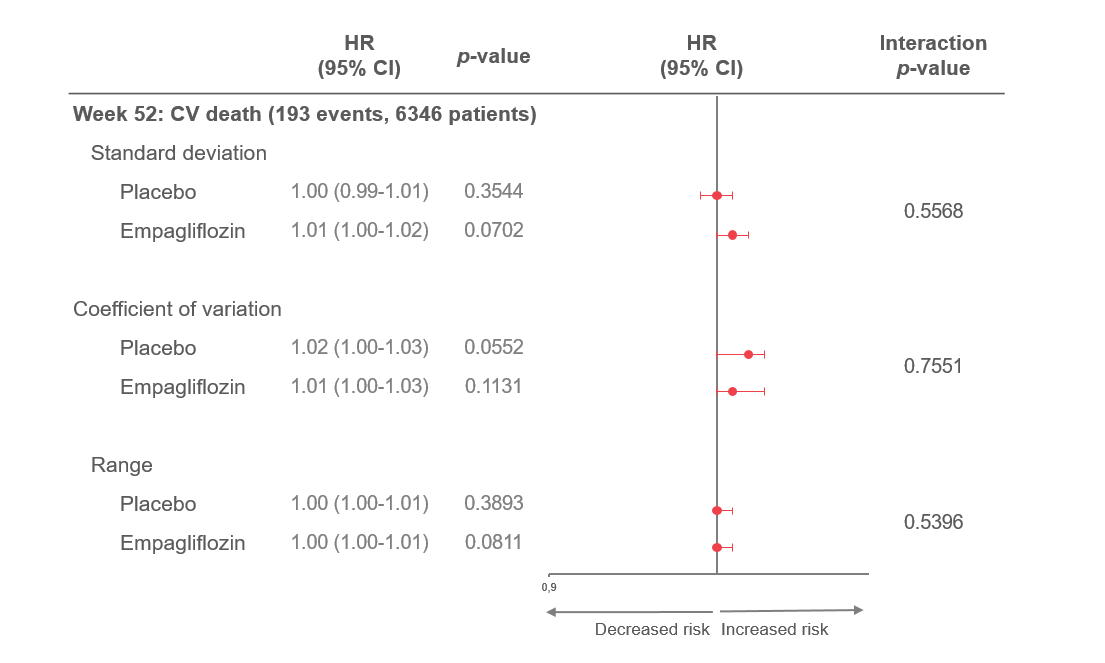


Only patients still at risk for CV death at week 28 (week 52, resp.) and with at least 2 post-baseline fasting blood glucose measurements up to week 28 (week 52, resp.) included. (466 and 674 patients were excluded from the 28 and 52 week analyses, respectively, due to lacking measurements). HRs are for a 1-unit (mg/dL) increase in fasting blood glucose variability. CV, cardiovascular; LM, landmark

Cox models include: age, sex, Hba1c, BMI, eGFR, geographic region, treatment, change in fasting blood glucose from baseline to week 4, blood glucose variability up to week 28 (week 52, resp.) and its interaction with treatment.

*blood glucose variability*treatment interaction.

**Additional file 1:Figure S5:** Association of fasting glucose variability variability by blood glucose as a continuous time-dependent co-variate and CV death in the treatment groups separately, by landmark analysis starting at week 4


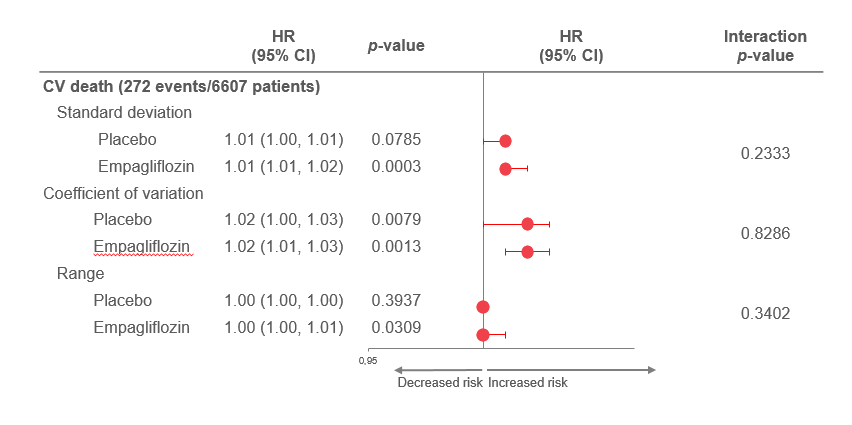


Only patients with least 2 pre-treatment and 2 post-baseline fasting blood glucose measurements included (413 patients were excluded due to lack of sufficient measurements). HRs are for a 1-unit mg/dL) increase in blood glucose variability.

CV, cardiovascular.

Cox models include: baseline age, sex, baseline Hba1c, baseline BMI, baseline eGFR, geographic region, treatment, change in fasting blood glucose from baseline to week 4, blood glucose variability as time-dependent covariate and its interaction with treatment.

* blood glucose variability*treatment interaction
